# Soluble Glycoprotein VI Levels Assessed Locally within the Extra- and Intracerebral Circulation in Hyper-Acute Thromboembolic Stroke: A Pilot Study

**DOI:** 10.3390/biomedicines12102191

**Published:** 2024-09-26

**Authors:** Andreas Starke, Alexander M. Kollikowski, Vivian Vogt, Guido Stoll, Bernhard Nieswandt, Mirko Pham, David Stegner, Michael K. Schuhmann

**Affiliations:** 1 Rudolf Virchow Center for Integrative and Translational Imaging, Julius-Maximilians-Universität Würzburg (JMU), 97080 Würzburg, Germany; andreas.starke@medizin.uni-leipzig.de (A.S.); bernhard.nieswandt@uni-wuerzburg.de (B.N.); david.stegner@uni-wuerzburg.de (D.S.); 2 Department of Neuroradiology, University Hospital Würzburg, 97080 Würzburg, Germany; kollikowsk_a@ukw.de (A.M.K.); pham_m@ukw.de (M.P.); 3 Department of Neurology, University Hospital Würzburg, 97080 Würzburg, Germany; vogt_v@ukw.de; 4 Institute for Experimental Biomedicine, University Hospital Würzburg, 97080 Würzburg, Germany; stoll_g@ukw.de

**Keywords:** blood platelets, GPVI, stroke, biomarker, carotid artery stenosis, thromboembolism

## Abstract

**Background:** Severe acute ischemic stroke (AIS) is mainly caused by thromboembolism originating from symptomatic carotid artery (ICA) stenosis or in the heart due to atrial fibrillation. Glycoprotein VI (GPVI), a principal platelet receptor, facilitates platelet adherence and thrombus formation at sites of vascular injury such as symptomatic ICA stenosis. The shedding of GPVI from the platelet surface releases soluble GPVI (sGPVI) into the circulation. Here, we aimed to determine whether sGPVI can serve as a local biomarker to differentiate between local atherosclerotic and systemic cardiac thromboembolism in AIS. **Methods:** We conducted a cohort study involving 105 patients undergoing emergency endovascular thrombectomy (EVT) for anterior circulation stroke. First, sGPVI concentrations were measured in systemic arterial plasma samples collected at the ipsilateral ICA level, including groups with significantly (≥50%) stenotic and non-stenotic arteries. A second sample, taken from the intracerebral pial circulation, was used to assess GPVI shedding locally within the ischemic brain. **Results:** Our analysis revealed no significant increase in systemic sGPVI levels in patients with symptomatic ≥ 50% ICA stenosis (3.2 [95% CI 1.5–5.0] ng/mL; *n* = 33) compared with stroke patients without significant ICA stenosis (3.2 [95% CI 2.3–4.2] ng/mL; *n* = 72). Additionally, pial blood samples, reflecting intravascular molecular conditions during collateral flow, showed similar sGPVI levels when compared to the systemic ICA samples in both groups. **Conclusions:** Our findings indicate that GPVI is not locally cleaved and shed into the bloodstream in significant amounts during hyper-acute ischemic stroke, neither at the level of symptomatic ICA nor intracranially during collateral blood supply. Therefore, sGPVI does not appear to be suitable as a local stroke biomarker despite strong evidence of a major role for GPVI-signaling in stroke pathophysiology.

## 1. Introduction

Severe acute ischemic stroke (AIS) is predominantly caused by thromboembolism originating either from the heart, particularly in cases of atrial fibrillation (AF), or from symptomatic internal carotid artery (ICA) stenosis [1,2,3]. These conditions differ fundamentally in their mechanisms of thromboembolic formation. In AF, thrombi develop within the left atrium due to local flow turbulences, primarily through the activation of the plasmatic coagulation cascade [4,5]. In symptomatic ICA stenosis (typically ≥ 50%), however, rupture of the atherosclerotic plaque exposes collagen to the bloodstream, triggering local platelet activation. This process involves initial interactions between the glycoprotein (GP) Ib-V-IX complex and the collagen-bound von Willebrand factor. Additionally, platelets are immobilized via direct binding to collagen, with GPVI functioning as the key receptor mediating these responses [3,6]. The transmembrane signaling receptor GPVI is expressed exclusively on platelets and megakaryocytes. Adherence of platelets to sites of vascular injury further triggers cellular activation, thus enabling integrin-mediated platelet adhesion and the formation of thrombi that can embolize into the cerebral vasculature. Interestingly, while GPVI levels are stable on resting or circulating platelets, platelet activation may elicit rapid metalloproteolytic cleavage, releasing soluble GPVI (sGPVI) into the circulation [7]. sGPVI has been established as a platelet-specific biomarker of activation with emerging clinical significance. For instance, systemic sGPVI levels were strongly associated with the onset of sepsis and were predictive of mortality in patients with thermal injuries from burns [8]. In addition, GPVI serves as the primary activating collagen receptor, further inducing the release of platelet α-granules, including the highly abundant platelet-derived neutrophil-activating chemokine, CXC motif ligand (CXCL) 4. Recently, we provided evidence of localized platelet activation within the collateral circulation of AIS patients, demonstrated by elevated cerebral ischemic plasma levels of CXCL4 compared with intraindividual systemic control samples [9]. Several animal studies have demonstrated that platelet GPVI plays a critical role in thrombosis, particularly in the context of ischemic stroke. Preclinical models of ischemic stroke have highlighted the therapeutic potential of targeting GPVI. For example, depletion of GPVI in mice subjected to transient middle cerebral artery occlusion—a commonly used model of ischemic stroke—significantly reduced infarct volume and improved neurological outcomes. Additionally, blocking platelet GPVI attenuated infarct growth during large vessel occlusion (LVO) prior to recanalization. These findings underscore the involvement of GPVI in the pathophysiology of AIS and suggest that GPVI inhibition could offer a therapeutic approach for AIS without increasing the risk of hemorrhage [10,11].

In the present study, we examined whether arterial sGPVI concentrations are elevated at sites of ipsilateral symptomatic (≥50%) ICA stenosis in hyper-acute stroke, and whether they can discriminate between underlying stroke pathophysiology. We measured sGPVI levels within the extracranial arterial circulation at the ipsilateral ICA site in a cohort of 105 stroke patients with LVO undergoing endovascular thrombectomy (EVT) [12]. Moreover, we studied sGPVI concentrations in the intracranial vasculature within the pial collateral circulation prior to EVT in this cohort as described by [13] to further explore the role of GPVI signaling and shedding in local ultra-early stroke pathophysiology [14] and to assess the utility of sGPVI as an intravascular cerebral biomarker. In addition, CXCL4 levels were assessed.

## 2. Materials and Methods

### 2.1. Patients and Blood Samples

We studied 105 patients who underwent emergency endovascular thrombectomy (EVT) for acute large vessel occlusion (LVO) stroke of the anterior circulation. Access for EVT was obtained using the modified Seldinger technique by a transfemoral approach. Recanalization of the predefined target lesions was preceded by microcatheter navigation (Neuroslider 27 or 21; Acandis, Pforzheim, Germany) into the mid-insular middle cerebral artery M2 segment. For reasons of variable vascular anatomy, the choice of a distinct microcatheter was at the discretion of the operator. After microcatheter positioning and discarding the respective microcatheter-specific dead space volume, a sample of 1 mL of cerebral ischemic arterial blood was drawn using citrate–phosphate–dextrose–adenine-1 (CPDA) as an anticoagulant [13]. Additionally, systemic arterial samples were drawn at the level of the cervical internal carotid artery (ICA) in stroke patients with ≥50% symptomatic ICA stenosis (*n* = 33) and in those without significant ICA pathology (*n* = 72). After obtaining the samples, centrifugation was performed to obtain cell-free plasma. Plasma was aliquoted, and promptly stored at −20 °C to preserve its integrity. The assessment of soluble GPVI was performed in all 105 patients, while residual plasma samples for quantifying CXCL4 were available for 91 patients (28 with and 63 without significant ICA pathology). For a portion of the cohort without significant ICA pathology, CXCL4 concentrations have been previously reported [9].

Inclusion criteria for the assessment of soluble GPVI and CXCL4 levels during AIS in the subgroup with no significant ICA pathology were as follows:

(1) AIS with severe neurological baseline deficit qualifying for EVT and (2) periprocedural (invasive angiographic) confirmation of LVO of the following sites: distal ICA (ICA-T), MCA M1 segment, or proximal M2 segment, respectively. Patients were excluded for the following reasons: (1) proven bilateral or multifocal LVO other than defined; (2) angiographically proven residual or restored antegrade blood flow; (3) any deviation from the interventional, sampling, and pre-processing protocols; (4) LVO in conjunction with either ≥ 50% cervical ICA stenosis or ICA dissection; and (5) intraprocedural percutaneous transluminal angioplasty (PTA) or stent implantation.

Patient inclusion criteria for the assessment of soluble GPVI and CXCL4 levels during AIS in the subgroup with significant ICA pathology were as follows:

(1) AIS with severe neurological baseline deficit qualifying for EVT; (2) invasive angiographic confirmation of LVO of the following sites: distal ICA (ICA-T), MCA M1 segment, or proximal M2 segment; and (3) LVO in conjunction with either ≥ 50% cervical ICA stenosis or ICA dissection, and intraprocedural percutaneous transluminal angioplasty (PTA) or stent implantation. Patients were excluded for the following reasons: (1) proven bilateral or multifocal LVO other than defined; (2) angiographically proven residual or restored antegrade blood flow; (3) any deviation from the interventional, sampling, and pre-processing protocols.

### 2.2. Enzyme-Linked Immunosorbent Assay

To assess sGPVI and CXCL4 plasma levels, patient samples were analyzed using commercially available sandwich enzyme immunoassay ELISA kits following the manufacturer’s instructions outlined in the product insert: GPVI, Sigma-Aldrich (#RAB1495, Taufkirchen, Germany); CXCL4, Thermo Scientific (#EHPF4, Dreieich, Germany). Measurements were conducted with a TECAN Infinite 200 PRO reader.

### 2.3. Study Approval

This observational study was approved by the local ethics committee of the Medical Faculty of the University of Würzburg, Germany (approval # 135/17). Written informed consent was obtained from all participants or their legal representatives.

### 2.4. Statistics

Statistical analyses were performed using GraphPad Prism 9 (version 9.3.1, GraphPad Software, San Diego, CA, USA). Data are presented as scatter dot plots with median. The normal distribution of datasets was tested using the D’Agostino-Pearsons test. The Wilcoxon rank-sum test or Mann–Whitney-U test was performed to test for significance. *p*-values < 0.05 (two-sided) were considered statistically significant.

## 3. Results

### 3.1. Comparison of Systemic sGPVI Concentrations in Patients with and without a Significant ICA Stenosis

In total, our analysis encompassed 210 plasma samples from 105 patients experiencing LVO in the anterior circulation. Among these, 33 patients (31.4%) exhibited an ipsilateral symptomatic ≥ 50% cervical ICA stenosis or dissection, while 72 patients exhibited no significant ipsilateral ICA stenosis. The cohort was predominantly male (66.7%) with an average age of 74.9 years (range: 72.6–77.2). A subset of the patients had a history of atrial fibrillation, with 49 patients (46.7%) presenting this condition. Additionally, antithrombotic medication was noted in 49 patients (46.7%), while 42 patients (40.0%) were administered alteplase prior to EVT as part of their routine medical management. A synopsis of the patients’ characteristics is shown in Table 1.

**Figure 1:**
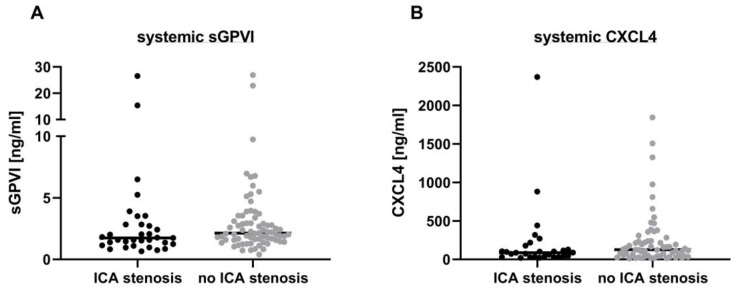
Concentration of sGPVI (**A**) and CXCL4 (**B**) in arterial plasma samples of patients with large-vessel ischemic stroke in the anterior circulation. Blood samples were obtained from the internal carotid artery (systemic) in patients with either ≥ 50% cervical ICA stenosis or ICA dissection (depicted by black points) and patients without significant ICA stenosis and most likely cardiogenic thromboembolism (depicted by grey points), immediately following EVT. The data are presented as a scatter dot plot with the median indicated. A Mann–Whitney-U test was performed to test for significance.

At the outset, our investigation focused on discerning potential variations in systemic arterial sGPVI concentrations among patients presenting with symptomatic ipsilateral (≥50%) ICA stenosis compared with those who did not suffer from a significant atherosclerotic ICA pathology and whose thromboembolism most likely originated in the heart. Upon analysis of systemic arterial plasma samples obtained immediately post-EVT, we found no statistically significant disparities in overall sGPVI concentrations between these patient subgroups (ICA stenosis: 3.2 [95% CI 1.5–5.0] ng/mL vs. no ICA stenosis: 3.2 [95% CI 2.3–4.2] ng/mL, *p* = 0.11; Figure 1A). In addition, CXCL4 levels were assessed, and again no statistically significant differences became apparent between the subgroups (ICA stenosis: 215.4 [95% CI 37.93–392.8] ng/mL vs. no ICA stenosis: 247.0 [95% CI 157.5–336.6] ng/mL, *p =* 0.19; Figure 1B).

### 3.2. Local sGPVI Concentrations in Cerebral Pial Blood Samples under LVO before EVT

There is strong evidence for platelet activation within collateral blood vessels maintaining residual blood flow within the ischemic territory before EVT [14]. This activation includes the release of chemokines from platelet alpha granules, such as CXCL4 [9]. 

**Figure 2:**
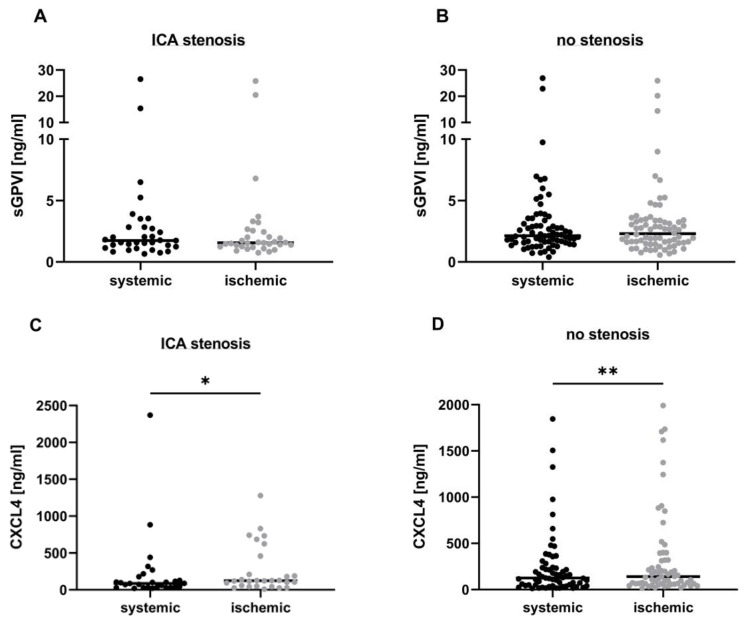
Local sGPVI and CXCL4 concentrations distal to cerebral artery occlusion in hyper-acute stroke. Local systemic (depicted by black points) and ischemic (depicted by grey points) plasma levels of sGPVI and CXCL4 in patients with either ≥50% cervical ICA stenosis or ICA dissection (**A**,**C**) and patients without significant ICA pathology (**B**,**D**). The data are presented as a scatter dot plot with the median indicated. A Wilcoxon rank-sum test was performed to test for significance. * *p*-value < 0.05; ** *p*-value < 0.01.

To evaluate whether this local platelet activation entails the shedding of GPVI, we quantified sGPVI levels in pial blood samples and compared them with extracranial (systemic) concentrations at the level of the ICA within the same individuals. Our analysis revealed no differences between systemic and ischemic plasma samples, neither in patients with ICA stenosis or dissection (systemic: 3.2 [95% CI 1.5–5.0] ng/mL vs. ischemic: 3.2 [95% CI 1.3–5.1] ng/mL, *p* = 0.28; Figure 2A) nor in the non-stenosis group (systemic: 3.2 [95% CI 2.3–4.2] ng/mL vs. ischemic: 3.3 [95% CI 2.4–4.2] ng/mL, *p* = 0.41; Figure 2B). In contrast, the analysis of CXCL4 plasma concentrations revealed significant differences between systemic and ischemic samples in both patient subgroups (ICA stenosis or dissection; systemic: 215.4 [95% CI 37.93–392.8] ng/mL vs. ischemic: 263.8 [95% CI 139.3–388.2] ng/mL, *p* = 0.035 (Figure 2C); non-stenosis group; systemic: 247.0 [95% CI 157.5–336.6] ng/mL vs. ischemic: 330.7 [95% CI 211.7–449.7] ng/mL, *p* = 0.001 (Figure 2D)).

These findings suggest that local GPVI shedding into the bloodstream is not substantially elevated in the ischemic brain before EVT, even in the presence of significant ICA stenosis or dissection. Despite strong evidence of platelet activation within the ischemic territory, sGPVI levels do not differ significantly in local cerebral pial blood compared with systemic arterial blood.

## 4. Discussion

This study evaluated the utility of sGPVI as a local biomarker in hyper-acute ischemic stroke. We examined two different vascular compartments: (i) the ipsilateral ICA, recognized as a critical site of emerging thromboembolism in the context of symptomatic ICA stenosis [3] and (ii) the intracerebral pial vasculature. The pial circulation sustains residual collateral blood flow crucial for maintaining perfusion during LVO prior to EVT but also contributes to a detrimental platelet-driven inflammatory response under conditions of ischemia/hypoxia, which involves GPVI signaling [9,14]. 

Thromboembolism in patients with symptomatic ICA stenosis is mainly caused by plaque rupture and ensuing exposition of collagen to the circulation [2], which leads to local platelet adhesion involving GPVI as shown in human atheromatous plaques [6,15]. Accordingly, Revacept, a competitive antagonist of GPVI, was applied to patients with high-grade symptomatic ICA stenosis in a recent phase 2 trial to prevent recurrent stroke, but only a combined endpoint of neurological and cardiological events and death reached statistical significance [16]. Despite evidence of local GPVI signaling in the destabilization and rupture of atherosclerotic plaques [3,6], we did not observe differences in systemic arterial sGPVI levels between patients with an ipsilateral ≥ 50% symptomatic ICA stenosis or dissection and stroke patients without a significant ICA stenosis in our present sample. At first glance, this contrasts with findings from Al-Tamini et al., who reported overall increased sGPVI levels in systemic venous plasma samples of acute stroke patients when compared with matched control subjects [17]. Importantly, our analytic approach differed significantly as it utilized arterial blood samples directly from the sites of vascular injury, e.g., the carotid artery, and from pial collaterals provisionally nourishing the ischemic brain under LVO before EVT. This method allowed us to specifically address compartmental GPVI shedding reflecting local platelet responses within the arterial system rather than cross alterations within the venous system. Under LVO before recanalization, pial collaterals maintain some residual retrograde blood flow within the ischemic territory. During EVT, the vessel occluding thrombus is routinely penetrated by a microcatheter, which allows retrieval of minute amounts of blood from the secluded ischemic brain territory before thrombus retraction [13,18]. By analyzing these arterial blood samples, we have shown that there is an accumulation of leukocytes, mainly neutrophils, within this vascular compartment compared with the systemic circulation at the level of the ICA. This local intravascular inflammatory response is partly driven by the chemokines CXCL4 and CXCL7 and the danger-associated molecular pattern HMGB1, which are all released by alpha granules of activated platelets [9,19,20]. Importantly, platelet granule release depends on platelet activation, such as in response to GPVI signaling, but does not mandatorily involve GPVI shedding. We have shown in experimental stroke that the absence of GPVI can mitigate infarct progression in mice before and after recanalization [11]. Based on these findings, we hypothesized that sGPVI may be used as a local intravascular biomarker for platelet activation in the ischemic brain, but we did not detect overall differences between the paired plasma samples taken at the ICA level compared with the intracerebral pial compartment samples. This suggests that GPVI is not a promising biomarker, either because it is not locally cleaved and shed into the bloodstream, or due to its rapid clearance once shed. In contrast, CXCL4 levels are also elevated in patients with ICA stenosis, extending our previous findings and further supporting the concept of local platelet activation within the intracerebral vasculature [9].

A particular strength of our study is the presentation of comparative direct human cerebral data obtained during stroke emergency care. There is a high interest in the development of biomarkers for disclosing the underlying stroke etiology and, even more importantly, for the early prediction of outcomes to guide treatment decisions and design add-on-therapies to thrombolysis and/or EVT [21]. Accordingly, using the same approach, we recently showed that levels of neutrophil-derived matrix metalloproteinase-9 within the pial blood vessels predicted bleeding complications and outcomes in AIS even before EVT was performed [22]. In a further search for mechanism-based biomarkers, our present data provide evidence that sGPVI appears not to be suitable as a local stroke biomarker despite the fact that GPVI signaling is critically involved in the pathophysiology of AIS and has emerged as a novel therapeutic target currently under clinical testing [23]. Limitations of our study relate to the limited sample size and its observational design and explorative character, which requires confirmation in larger cohorts. Moreover, since blood is taken during an emergency procedure, no baseline samples for comparison are available.

## Figures and Tables

**Table 1:** Demographic, clinical, and laboratory characteristics of patients (*n* = 105).

Subgroup with no Significant ICA Pathology					
**Variable**	**%**	**Mean**	**95% CI**	**Min.–Max.**	***n***
*Demographics*					
Age [years]		76.3	73.6–79.0	43–94	72
Male sex	66.7				48
*Medical history and medication*					
Atrial fibrillation	55.6				40
Antithrombotic medication	55.6				40
Alteplase treatment	38.9				28
*sGPVI concentration (ng/mL)*					
Systemic		3.2	2.3–4.2	0.4–26.9	72
Ischemic		3.3	2.4–4.2	0.6–26.0	72
*CXCL4 concentration (ng/mL)*					
Systemic		247.0	157.5–336.6	12.8–1845.0	63
Ischemic		330.7	211.7–449.7	15.2–1991.0	63
**Subgroup with ≥50% ipsilateral symptomatic ICA stenosis**					
*Demographics*					
Age [years]		71.7	67.3–76.0	49–90	33
Male sex	66.7				22
*Medical history and medication*					
Atrial fibrillation	27.3				9
Antithrombotic medication	27.3				9
Alteplase treatment	42.4				14
Stenting/PTA	60.6				20
ICA dissection	15.2				5
*sGPVI concentration (ng/mL)*					
Systemic		3.2	1.5–5.0	0.7–26.6	33
Ischemic		3.2	1.3–5.1	0.8–25.8	33
*CXCL4 concentration (ng/mL)*					
Systemic		215.4	37.93–392.8	20.21–2369.0	28
Ischemic		263.8	139.3–388.2	9.350–1278.0	28

## Data Availability

The original contributions presented in the study are included in the article, further inquiries can be directed to the corresponding author.
